# Deep Learning Methods for Underwater Target Feature Extraction and Recognition

**DOI:** 10.1155/2018/1214301

**Published:** 2018-03-27

**Authors:** Gang Hu, Kejun Wang, Yuan Peng, Mengran Qiu, Jianfei Shi, Liangliang Liu

**Affiliations:** ^1^College of Automation, Harbin Engineering University, Harbin 150001, China; ^2^College of Business, Anshan Normal University, Anshan 114007, China; ^3^760 Research Institute of China Shipbuilding Industry, Liaoning, Anshan, China

## Abstract

The classification and recognition technology of underwater acoustic signal were always an important research content in the field of underwater acoustic signal processing. Currently, wavelet transform, Hilbert-Huang transform, and Mel frequency cepstral coefficients are used as a method of underwater acoustic signal feature extraction. In this paper, a method for feature extraction and identification of underwater noise data based on CNN and ELM is proposed. An automatic feature extraction method of underwater acoustic signals is proposed using depth convolution network. An underwater target recognition classifier is based on extreme learning machine. Although convolution neural networks can execute both feature extraction and classification, their function mainly relies on a full connection layer, which is trained by gradient descent-based; the generalization ability is limited and suboptimal, so an extreme learning machine (ELM) was used in classification stage. Firstly, CNN learns deep and robust features, followed by the removing of the fully connected layers. Then ELM fed with the CNN features is used as the classifier to conduct an excellent classification. Experiments on the actual data set of civil ships obtained 93.04% recognition rate; compared to the traditional Mel frequency cepstral coefficients and Hilbert-Huang feature, recognition rate greatly improved.

## 1. Introduction

Deep learning is a new area of machine learning research aimed at establishing a neural network that simulates human brain analysis and learning. The concept of deep learning was proposed by Hinton in 2006 [1]. In recent years, deep learning has drawn wide attention in the field of pattern analysis and has gradually become the mainstream method in the fields of image analysis and recognition and speech recognition [2]. In recent years, some people apply it to the speech signal denoising [3] and the dereverberation problem [4]. Convolutional Neural Network (CNN) is one of the core methods in depth learning theory. CNN can be classified as deep neural network (DNN) but belongs to supervised learning method. Y. LeCun proposed CNN is the first real multilayer structure learning algorithm, which uses space relative relationship to reduce the number of parameters to improve training performance, and has achieved success in handwriting recognition. Professor Huang Guangbin from Nanyang Technological University first proposed extreme learning machine in 2004. Extreme learning machine (ELM) has been proposed for training single hidden layer feed forward neural networks (SLFNs). Compared to traditional FNN learning methods, ELM is remarkably efficient and tends to reach a global optimum. In this paper, CNN and ELM methods are introduced into underwater acoustic target classification and recognition, and a underwater target recognition method based on depth learning is proposed. In view of the prominent performance of convolutional neural network in speech recognition and its frequent usage in speech feature extraction, the convolutional neural network is used to extract the features of underwater ship's sound signal, and the corresponding network model and parameter setting method are given. This method is compared with Support Vector Machine (SVM) [5] and* k*-nearest neighbors (KNN) [6], Hilbert-Huang Transform (HHT) [7], and Mel frequency cepstral coefficients (MFCC) [8] methods. The experimental results show that underwater target recognition based on depth learning has a higher recognition rate than the traditional method. Then ELM fed with the CNN features is used as the classifier to conduct an excellent classification. Experiments on the actual data set of civil ships obtained 93.04% recognition rate; compared to the traditional Mel frequency cepstral coefficients and Hilbert-Huang feature, recognition rate greatly improved.

## 2. Related Work

The current development of passive sonar high-precision underwater target automatic identification method to prevent all types of water targets in the raid is to strengthen the urgent task of the modern war system. A series of theoretical methods and technical means involved in the automatic identification of underwater targets can be applied not only to national defense equipment research but also to marine resources exploration, marine animal research, speech recognition, traffic noise recognition, machine fault diagnosis, and clinical medical diagnosis field.

In the 1990s, researchers of all countries applied artificial neural network into underwater target recognition system. The methods such as power spectrum estimation, short-time Fourier transform, wavelet transform, Hilbert-Huang transform, fractal, limit cycle, and chaos [8–11] failed to fully consider the structure features of sound signal and the features extracted by such methods have prominent problems, such as worse robustness and low recognition rate.

In 2006, a paper published on* Science, *the world top level academic journal, by Geoffrey Hinton, machine learning master and professor of University of Toronto and his student Ruslan, aroused the development upsurge [12] of deep learning in research field and application field. Since then, many researchers began working on deep learning research. In 2009, Andrew Y. Ng, etc. [13] extracted once again the features of spectrogram by using convolutional deep belief networks, and all of the results derived from use of extracted features to multiple voice recognition tasks are superior to the ones of the system which recognizes by taking Mel frequency cepstrum coefficient directly as a feature. In 2011, Hinton used restricted Boltzmann machine [14] to learn the waveform of original voice signal to obtain the distinguishable advanced features. The experiment shows that such method is better than the traditional Mel frequency spectrum feature in performance. In 2012, four major international scientific research institutions summed up the progress made by deep learning in sound recognition task [15] and pointed out in the paper that the experiments show the effect of deep neural network is better than the one of traditional Gaussian mixed model. In 2013, Brian Kingsbury, researcher of IBM, and several other persons [16] took the logarithmic Mel filter coefficient as the input of deep convolution network and further extracted the “original” features (Mel filter coefficient), and the experiment shows that the recognition rate has a relative increase of 13–30% compared to the traditional Gaussian mixed model and has a relative increase of 4–12% compared to deep neural networks. The research of Palaz et al‎. [17] shows that the system of using original voice signal as the input of convolutional neural networks to estimate phoneme conditional probability has achieved similar or better results than the TIMIT phoneme recognition task of the standard hybrid type HMM/ANN system. In 2014, Palaz et al. [17] put forward the convolutional neural networks of restriction right sharing mechanism and once again achieved amazing results. In 2015, three artificial intelligent masters, LeCun et al. [18], jointly published an overview titled “deep learning” on* Nature*, giving a comprehensive introduction on the theory of deep learning. Nowadays, deep learning has become the research hot spot of the world. Considering the outstanding performance of convolutional neural networks in voice recognition, this article uses convolutional neural networks to conduct feature extraction and recognition to underwater ship voice signal.

## 3. Deep Convolutional Neural Networks

Under the inspiration of the structure of visual system, Convolutional neural networks (CNN) are developed. The first convolutional neural networks calculation model is put forward in the neural recognizer of Fukushima, which applies the neuron with the same parameters to different positions of the upper layer of neural networks based on partial connection and hierarchical organization image transformation, thus generating a kind of translation invariant neural network structural form. Afterwards, based on such thought, Abdel-Hamid et al. [19] designed and trained convolutional neural network by using error gradient and demonstrate an outstanding performance in handwriting character recognition task. The convolutional neural networks can be regarded as kind of transformation of standard neural networks, which introduce a kind of so called convolutional layer and sampling layer structure rather than using the whole connection structure like the traditional neural networks.

**Figure 1:**
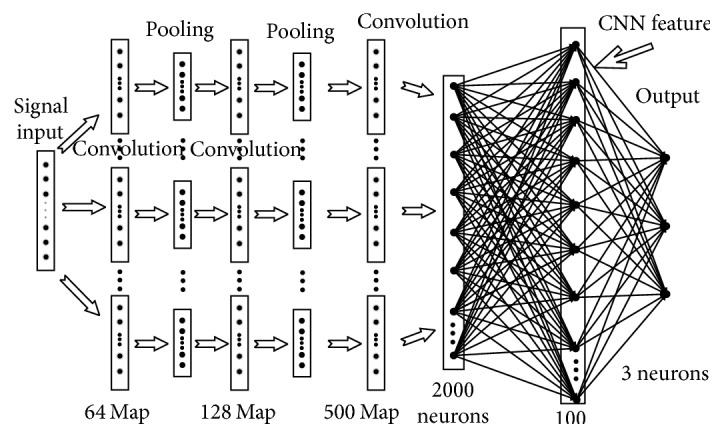
Structure chart of deep convolutional networks.

Deep convolutional neural networks refer to the networks containing two layers or more than two layers of convolutional layer and sampling layer in the whole network. Each convolutional layer contains several different convolvers (filters) and these convolvers make observation to all partial features of the signal. Next to convolutional layer usually comes sampling layer, and sampling layer reduces the input node numbers of next layer by making the sampling of fixed window length to the output node numbers of the convolutional layer so as to control the complexity of the model. Then, convolution and sampling are made again to the output features of sampling layer, thus forming a kind of layered feature extractor and output of each layer can be regarded as an advanced expression form (i.e., advanced feature) of the “original” voice signal. Though convolutional neural networks also can realize classification of signal, its classification effect mainly relies on the artificial neural networks classifier formed by whole connection layer, while the artificial neural networks classifier has the problems such as worse generalization ability and local optimum, so this article only makes feature extraction by using convolutional networks. The structure of feature extraction of the convolutional neural networks herein is shown in Figure 1.

Different from Multilayer Perception (MLP) and other neural network structures, CNN introduces three important concepts: partial connection, pooling, and weight sharing. Hereunder, we will introduce in detail the CNN structure in sound recognition and how to understand such three important concepts of CNN in sound recognition.

### 3.1. Partial Connection and Weight Sharing

**Figure 2:**
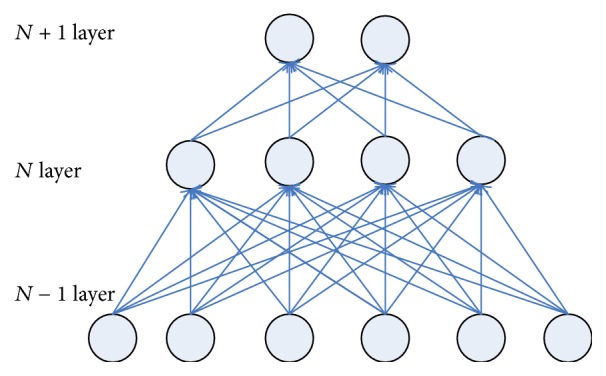
Sketch figure of full connection neural networks.

**Figure 3:**
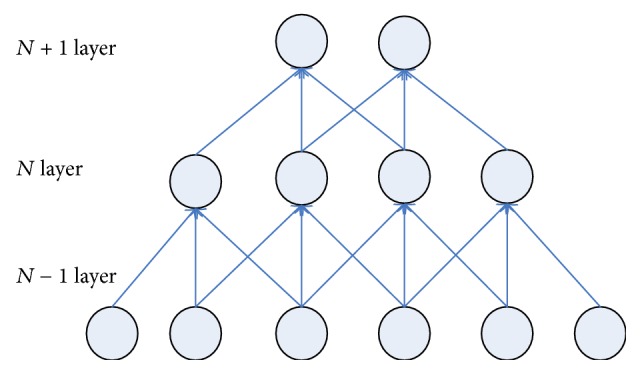
Sketch figure of partial connection.

In traditional BP network, the neurons in each layer show a one-dimensional linear array structure, with neuron nodes between each layer fully connected, while, in convolutional neural networks, neurons of each layer no longer adopt full connection form, with neurons at lower layer connected partially with some neurons at upper layer (partial connection). Figures 2 and 3, respectively, represent fully connected neural networks and partially connected neural networks.

As far as Figures 2 and 3 are concerned, the number of weights that need to be trained between fully connected neural network *N* − 1 layer and *N* layer is 24 (4 × 6) while the number of weights that need to be trained between partially connected neural networks *N* − 1 layer and *N* layer is only 12 (4 × 3). In this case, the number of training parameters only reduces slightly, but for a large neural network, each layer contains hundreds of neurons; the advantages of partial connection will become more prominent.

Weight sharing mechanism can further reduce the number of parameters that need to be trained in the networks. As shown in Figure 3, is concerned, if the weight sharing mechanism is used, the four groups of weight in the figure are the same (with each group assigned 3 weights, i.e., the convolutional kernel as mentioned hereunder); then there are only three weights that need to be trained between layer *N* − 1 and layer *N*, thus significantly reducing network parameters and reducing the complexity of the model.

### 3.2. Build the Input Features of Convolutional Networks

**Figure 4:**
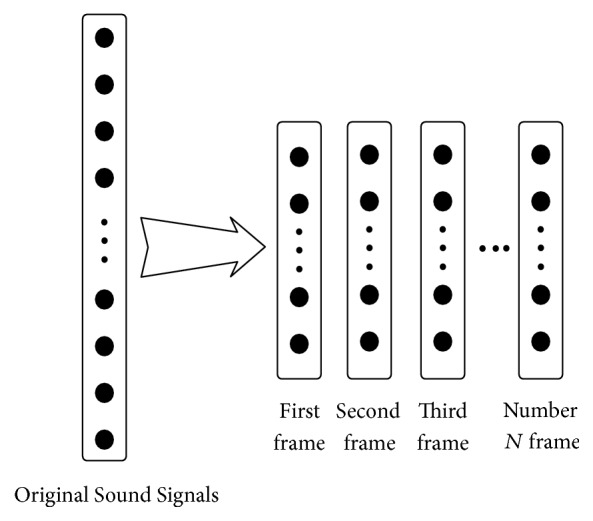
Build the input features of convolutional networks.

The most commonly used feature in sound recognition is Mel frequency cepstrum coefficient (MFCC), which is widely applied in semantic recognition and voice recognition; however its recognition rate is not ideal. In the course of extracting the features of MFCC, the feature extraction is made only according to experiences, not considering inner link of signals themselves. In order to rationally use all of the information of the signal and extract a more suitable feature, this article adopts the extraction method of extracting self-adaptively the advanced features of signal through multilayer convolution by taking the original sound signal after overlapping framing directly as the input feature figure of convolutional networks. The input feature sketch figure of convolutional networks is built in Figure 4.

As is shown in Figure 4, firstly, the input sound signal is a one-dimensional signal. Different from traditional method, this article causes the original sound signal to be processed directly by framing (framing method is overlapping segmentation, frame length is 170 ms, and frame shift is 10 ms), with each frame after framing as an input sample of convolutional networks, that is, as the input feature figure of deep convolutional networks.

### 3.3. Convolutional Layer

As the convolutional layer of convolutional neural networks has been defined with corresponding receptive field, for the signal of each neuron of convolutional layer, only those transmitted from their receptive field can be accepted. Each neuron in convolutional layer is only partially connected to input of current layer, which is equivalent to using a convolutional kernel to perform ergodic convolution to the input feature figure of current layer. Moreover, as one kind of convolutional kernel can only observe limited information, in practical application, multiple different convolvers are usually used to observe from different perspectives so as to acquire more amount of information.

In convolutional layer, the following two circumstances may occur: one is that there is only one input feature figure of convolutional layer; another is there are many input feature figures of convolutional layer. Given the above different circumstances, in order to make a better explanation, this article performs a detailed argumentation by combining the figures and texts.

**Figure 5:**
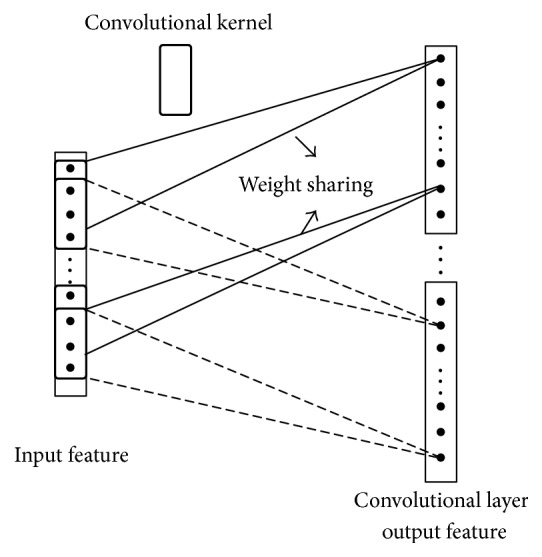
Sketch figure of convolution process of single input feature figure.

As is shown in Figure 5, there is only one input feature figure of convolutional layer (the process from input layer to convolutional layer), where the value of convolution result coming from the convolution to certain part of the feature figure by convolutional kernel and output by nonlinear function is the activated value of corresponding neuron in the feature figure of convolutional layer, and a convolutional layer output feature figure can be acquired by traversal input feature figure with convolutional kernel, and such output feature figure is equivalent to another kind of expression form to upper layer of input feature, that is, a feature learnt through convolutional neural networks. Many feature figures can be acquired from convolutional layer by traversal input feature figure with multiple convolutional kernels, by which, signals with different features can be extracted (different connection lines in the figure represent the connection of different convolutional kernels). The size and number of the convolutional kernels have an important impact on the performance of the whole network, and the key of deep convolutional networks is to learn the contents, size, and number of convolutional kernels and determine an appropriate convolutional kernel for adaptive feature extraction through learning mass data.

If the input of convolutional layer has only one feature figure, the output of each neuron in convolutional layer can be expressed by the following formula:

(1) $$\begin{array}{c} \alpha_{i}^{p}=\delta \left(\;\overset{H}{\underset{h=1}{\sum }}f_{h}^{p}\cdot x_{i+h-1}+b\;\right). \end{array}$$

In the formula, the size of convolutional kernels is *H∗*1 and is the element corresponding with line *h* of the number *p* of convolutional kernel; *x*_*i*_ is the activated value of input layer neuron connected with line *i* of neuron in feature figure of convolutional layer. *α*_*i*_^*p*^ is the activated value of the line *i* neuron in the number *p* convolution feature figure, and *δ* is the activated function of the networks, and *b* is bias.

**Figure 6:**
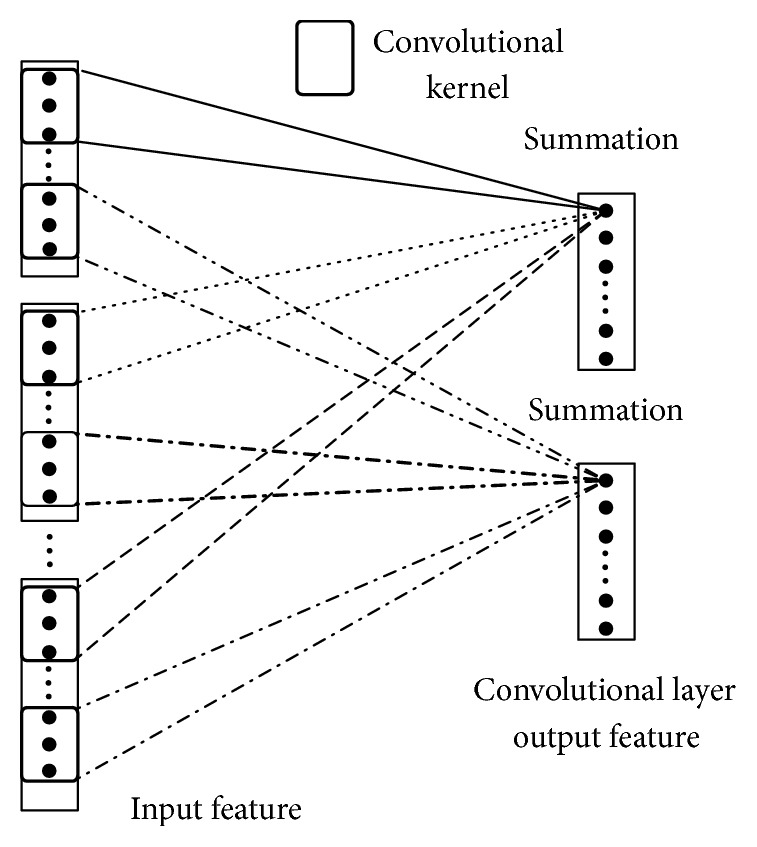
Convolution process sketch figure.

As is shown in Figure 6, there are many input feature figures of convolutional layer (i.e., the process from pool layer to convolutional layer, and different linetype in the figure represents different convolutional kernels), where the value output through nonlinear function by adding the convolution results coming from the convolution to certain part of all of the input feature figures by using multiple kernels is the activated value of the corresponding neuron in the output feature figure of convolutional layer, and an output feature figure of convolutional layer can be acquired by traversing many input feature figures with this group of convolutional kernels.

In case there are many input feature figures in the convolutional layer, the activated value of each neuron in number *p* feature figure in the convolutional layer can be expressed by the following formula:

(2) $$\begin{array}{c} \alpha_{i}^{p}=\delta \left(\;\overset{N}{\underset{k=1}{\sum }}\;\overset{H}{\underset{h=1}{\sum }}f_{h}^{k}\cdot x_{i+h-1}^{k}+b\;\right). \end{array}$$

In the above formula, *N* represents the number of input feature figures, *x*_*i*_^*k*^ represents the activated value of the neuron in number *k* input feature figure which connects with line *i* neuron in number *p* feature figure in convolutional layer, *f*_*h*_^*p*^ is the element corresponding with line *h* in number *p* convolutional kernel, *α*_*i*_^*p*^ is the activated value of neuron in line *i* in number *p* convolution feature figure, and *δ* is the activated function of the network and *b* is bias.

### 3.4. Pool Layer

**Figure 7:**
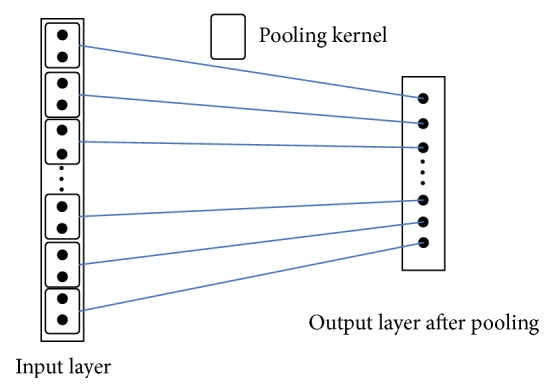
Sketch figure of mean value pooling.

Pool layer (also known as sampling layer) is not only a sampling processing to upper layer of feature figure, but also an aggregate statistics to features of different positions of upper feature figure. After sampling, not only can the models become less complicated to a large extent, but also overfitting can be reduced. Two ways often used in pool layer are mean value sampling and maximum value sampling. Mean value sampling method is to get the mean value in the neighboring small fields of upper layer of feature figure, which means value is taken as the activated value of the corresponding neuron in lower level feature figure. As the name suggests the maximum value sampling is to get the maximum value in the neighboring small fields of upper layer of input feature figure, where maximum value is the activated value of the corresponding neuron in lower level feature figure. In the experiment part, comparison experiment will be conducted to such two different pooling methods, and what is shown in Figure 7 is the mean value pooling process to the input feature figure by a 2*∗*1 pool kernel.

Mean value pooling can be expressed by formula (3):

(3) $$\begin{array}{c} \alpha_{i}=\frac{1}{L_{1}}\overset{L_{1}}{\underset{l_{1}=1}{\sum }}x_{\left(i-1\right)\cdot s+l_{1}}. \end{array}$$

In the formula, *α*_*i*_ is the output value of neuron after sampling, *x* is the input of neuron in corresponding sampling layer, *L*_1_ is the length of pool kernel, and *s* is the moving step size of the pool kernel.

## 4. Classifier of Extreme Learning Machine

In the single hidden layer feedforward neural network trained by BP algorithm, there are some main problems such as partial extreme value and training duration. In order to overcome these defects, professor Huang Guangbin of Nanyang Technological University firstly put forward the extreme learning machine [20, 21] algorithm in 2004. The prominent advantage of extreme learning machine is fast in training speed, which enables it to complete the training of feedforward neural network within several seconds or even less than a second, while the training of the traditional single hidden layer network based on back propagation algorithm usually needs several minutes, several hours or even several days. Another advantage of a learning machine is the enhanced generalization ability.

**Figure 8:**
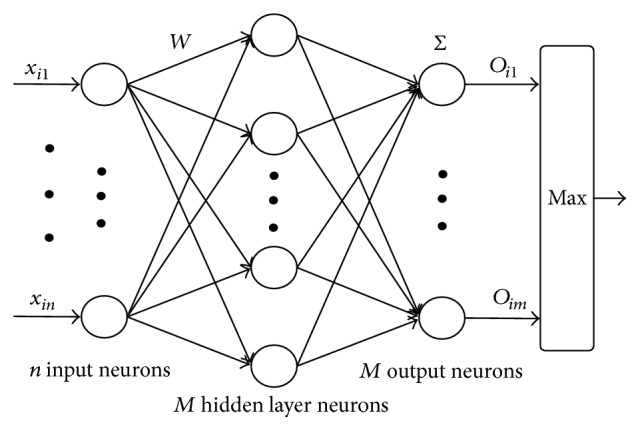
Extreme learning machine classifier.

Figure 8 is standard extreme learning machine classifier with hidden layer containing *M* neurons. According to the theory of Huang Guangbin, if the activated functions of hidden layer neurons are infinitely differentiable, and initialization weight and bias are entered at random, extreme learning machine can approach any sample with no error. That is to say for the given training sample (*X*_*i*_, *T*_*i*_)  *i* = 1,2,…, *N*, there into *X*_*i*_ = [*x*_*i*1_, *x*_*i*2_,…,*x*_*in*_]^*T*^ ∈ *R*^*n*^ and *T*_*i*_ = [*t*_*i*1_, *t*_*i*2_,…,*t*_*im*_]^*T*^ ∈ *R*^*m*^ have *β*_*j*_; *W*_*i*_ and *b*_*i*_ cause the formation of

(4) $$\begin{array}{c} \overset{M}{\underset{j=1}{\sum }}\beta_{j}f\left(\;w_{ij}\cdot x_{i}+b_{j}\;\right)=o_{i}=T_{i}\;i=1,2,\ldots ,N. \end{array}$$

Herein, *β*_*j*_ is the weight vector connecting number *j* neuron in hidden layer and all neurons in output layer, *o*_*i*_ is output vector of number *i* sample of single hidden layer feedforward network, *T*_*i*_ is the category label vector of number *i* sample, *w*_*ij*_ is the weight vector connecting number *i* sample and number *j* neuron in hidden layer, *b*_*j*_ is the bias of number *j* neuron in hidden layer, and *f*(·) is activated function. Formula (4) can be written as the matrix form as shown in formula (5), and *H*_*w*,*x*,*b*_ is called output matrix of hidden layer.

(5) Hw,x,bβ=T, β=β1Tβ2T⋮β1TβMT,  T=T1TT2T⋮T1TTNTN×M,

(6) $$\begin{array}{c} H_{w,x,b}=\left[\begin{array}{ccc} f\left(W_{1}\cdot X_{1}+b_{1}\right) & \cdots & f\left(W_{M}\cdot X_{1}+b_{M}\right)\\ \vdots & \ddots & \vdots \\ f\left(W_{1}\cdot X_{N}+b_{1}\right) & \cdots & f\left(W_{M}\cdot X_{N}+b_{M}\right) \end{array}\right]. \end{array}$$

What formula (5) expresses is a linear system. In case that the number of hidden layer neurons in the network is the same with the number of training sample, its output layer matrix is a square matrix, and it can be known from the two bold theories of Huang Guangbin that such square matrix is reversible; then the least square solution of such system is *β* = *H*^−1^*T*. However, in fact, the number of nodes in hidden layer usually is less than the number of samples, so the matrix of output layer is not a square matrix. The least square solution of such linear system is *β* = *H*^+^*T*, in which, *H*^+^ is the generalized inverse matrix of *H*_*w*,*x*,*b*_.

From the above analysis, we can see that the training process of the extreme learning machine classifier can be divided into the following three steps:

1. Initialize the input weight *W*_*i*_ and bias *b*_*i*_  (*i* = 1,2,…, *M*) of the network at random;
2. Calculate the output matrix *H*_*w*,*x*,*b*_ of the hidden layer and its generalized inverse matrix *H*^+^;
3. Calculate output weight *β* by formula *β* = *H*^+^*T*.

After the value of *β* is calculated, the training work of extreme learning machine classifier is finished. If a test sample *X* of an unknown label is given, classification can be made by a well trained extreme learning machine classifier, whose category can be obtained by the calculation of formula (7):

(7) $$\begin{array}{c} T_{M}\left(\;X\;\right)=H\left(\;X\;\right)\beta , \end{array}$$

in which, $H\left(X\right)=\left[\begin{array}{ccc} f(W_{1},b_{1},X) & \cdots & f(W_{M},b_{M},X) \end{array}\right]$ is response of hidden layer to *X*.

## 5. Experiment Results and Analysis

### 5.1. Experiment Conditions and Parameters Setting

**Figure 9:**
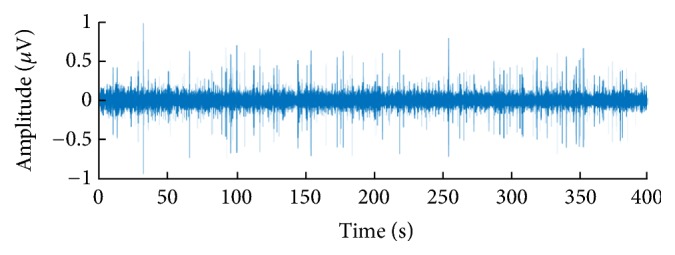
Time domain diagram of underwater noise.

**Figure 10:**
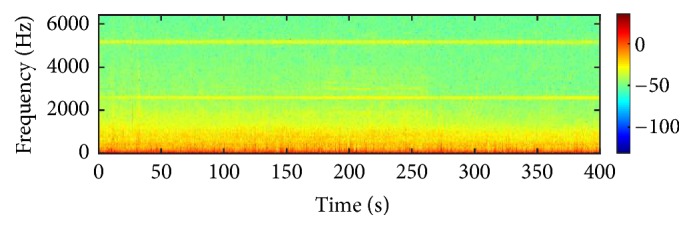
Frequency domain diagram of underwater noise.

All experiments mentioned in this article are completed on the server of the laboratory, and the configuration of the server is as follows: 64 bits win 7 operation system, 64 GB memory, 24 cores of CPU and K40 GPU of NVIDIA Company, is equipped for accelerated calculation. Software used in the experiments is the latest version of MATLAB 2015a, latest version of VS2013, and the version of CUDA is 6.5. All data of the experiment come from the actual civil ship data samples. The experimental data come from the civil ship data sample, which is the waveform data from the original ship target radiated underwater noise passed through the sonar into the ADC converter output.  Figure 9 shows a time domain diagram of the underwater acoustic signal of a civil boat sample and Figure 10 shows the frequency domain of the underwater acoustic signal of a civil boat sample. The colour scale represents energy and is measured in microvolts. Such dataset includes three kinds of civil ships, that is, small ship, big ship, and Bohai ferry, and the sampling place is the anchorage ground, and sampling frequency is 12800 Hz. In the experiment, eighty percent of each kind of samples are used as training set, and the rest twenty percent are used as test set.

The feature extraction process of CNN involves large quantity of parameters, and this article gets the optimal network structure as shown in Table 1 through numerous experiments.

In order to facilitate the programming realization, the full connection process is equivalent to using a convolutional kernel of 1*∗*1 to sum the convolution of all upper feature figures, and the number of feature figures equals to the number of neurons in current layer of full connection network. The first pool layer chooses maximum value pooling, and the second pool layer chooses mean value pooling. The combination of maximum value pooling and mean value pooling will achieve better effects. The typical parameter value choice and selection involved in neural network training process are shown in Table 2.

### 5.2. Experiment Analysis

In pooling process, there are two commonly seen pooling methods: mean value pooling and maximum value pooling. This article compares the two different pooling methods and the chosen classifiers are all the extreme learning machines. The experiment results are shown in Table 3.

Table 3 shows that choosing different pooling method in the pooling process has a big impact on the features extracted. The performance with both pool layers using mean value pooling is the worst one, while the combination of mean value pooling and maximum value pooling in the pooling process can achieve an ideal effect; besides, the effect with the last pool layer of the network using mean value pooling is superior to the one using maximum value pooling.

This article compares the traditional “manual features” (Mel frequency cepstrum coefficient feature and Hilbert-Huang transform feature) and the features “automatically” extracted by deep convolutional networks as used by this article. All the classifiers chosen in the experiment are extreme learning machine classifiers; however, number of neurons in hidden layer of extreme learning machine and the activated functions used by the learning machine are different. The experiment results are shown in Table 4.

The Table 4 experiment shows that the features obtained by extracting the features of original sound signals through deep learning are effective, and the ELM algorithm can be used to realize separation to civil ship sound signals, and the recognition rate on the test set can reach 93.04%. Compared with traditional MFCC features and the features obtained from Hilbert-Huang transform, the features obtained from deep learning are easier to be classified. As to the recognition rate, the rates obtained from features adopted by this article are 5%~10% higher than those obtained from traditional features, which is mainly because the traditional features are the ones generated “manually,” without fully considering the inner links of signal, while deep learning can realize “automatic” extraction of features.

Besides, comparison is made on different features of extreme learning machine classifier mentioned in the article and traditional classifier. In such experiment, number of hidden layer neurons of extreme learning machine is 40, and Tables 5 and 6, respectively, give the effects of the three different classifiers on classification and recognition of MFCC features and features obtained from deep learning.

In Table 6, both the training time and test time refer to mean time of single samples. It can be seen intuitionally from the table that though* K* neighboring classifier and SVM classifier can realize feature space division, the recognition rates of the two obviously are lower than the recognition rate of ELM classifier; what is more, training time and test time of the two far exceed the time used by ELM.

**Figure 11:**
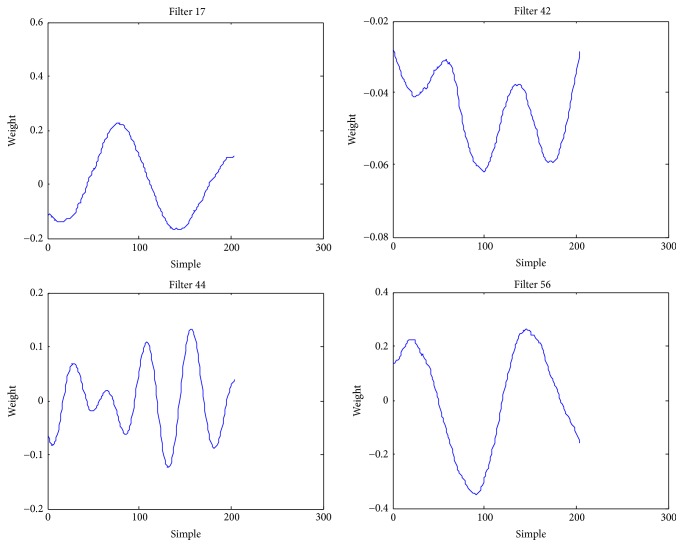
The coefficients of some convolution kernels.

The analysis made in the comparison experiment in Table 4 shows that the features obtained from “automatic” feature extraction using deep convolutional networks are superior to the traditional features generated “manually”; however, a model extracted by using neural network is an issue worthy of thinking. Making an analysis on all parameters in the network obviously is not possible; however, we can analyze and process the convolutional kernels learnt from convolutional layer.  Figure 11 gives part of the convolutional kernels obtained through learning in the first convolutional layer in the convolutional neural networks. The* X*-axis means the sequence number of the convolution kernel sequence, while the* Y*-axis means the value of the convolution kernel sequence. Apparently, these convolutional kernels serve as one and another band-pass filters and, respectively, respond to different frequency bands of transmission signals, and the convolutional kernels obtained from these learnings can be regarded as a matched filter. Through making research on the amplitude frequency features of the convolutional kernels obtained, we find that these filters are distributed nonlinearly and mostly located in low frequency band. It can be seen from Figure 11 that the useful components of the underwater noise signal of a civil ship are concentrated in the low frequency part of the frequency domain.

## 6. Conclusion

This article conducts feature extraction to original waveform of underwater sound signal by adopting deep convolutional neural networks and takes the extracted features as the input features of extreme learning machine classifier and realizes the classification and recognition to underwater sound signals. This article compares the feature extraction method set forth in this article and the traditional feature extraction methods and validates the effectiveness of the feature extraction methods used herein. Meanwhile, this article compares the classification effects and classification times of different classifiers at the classifier stage and highlights the advantages of the classifier used by this article in classification time and classification precision. Compared with the traditional MFCC and HTT, the recognition rate of 93.04% has been greatly improved on the actual civil ship data set. Experimental results show that CNN can be better used to extract the feature extraction and recognition of underwater target noise data.

## Figures and Tables

**Table 1:** CNN structure parameters.

Layer number	Type	Number of feature figures	Size of feature figures	Size of kernel	Length of step
(1)	Input layer	1	2176*∗*1	—	—
(2)	Convolutional layer	64	79*∗*1	204*∗*1	25
(3)	Sampling layer	64	39*∗*1	2*∗*1	2
(4)	Convolutional layer	128	28*∗*1	12*∗*1	1
(5)	Sampling layer	128	14*∗*1	2*∗*1	2
(6)	Convolutional layer	500	1*∗*1	14*∗*1	1
(7)	Full connection	2000	1*∗*1	1*∗*1	1
(8)	Full connection	100	1*∗*1	1*∗*1	1
(9)	Full connection	3	1*∗*1	1*∗*1	1

**Table 2:** Value choice and selection of typical parameters.

Parameters	Scope
Activated functions	ReLu
Size of learning rate	0.02~0.05
Decay rate of learning rate	0.0005/Batch
Number of batch processing samples (Batch)	200
Impulse magnitude	0.9

**Table 3:** Comparison of different pooling methods.

Frame size	Pooling method	Number of ELM hidden layer nodes	Recognition rate (%)
2176	Maximum value	40	92.88
2176	Mean value	40	90.80
2176	Maximum value-mean value	40	93.04
2176	Mean value-maximum value	40	91.65

**Table 4:** Comparison of classification effects of different features.

Feature types	Number of hidden layer neurons	ELM activated functions	Recognition rates (%)
MFCC	40	Sigmoid	84.64
	60	Sigmoid	84.48
	80	Sigmoid	84.41
	60	tanh	82.39

HHT	40	Sigmoid	81.06
	60	Sigmoid	82.34
	40	tanh	81.72
	60	tanh	82.04

Deep learning feature	40	Sigmoid	90.39
	60	Sigmoid	92.69
	20	tanh	92.40
	40	tanh	93.04
	60	tanh	92.29

**Table 5:** Comparison of performance of different classifiers (MFCC features).

Names of classifier	Training time (S)	Time of classification (S)	Recognition rate (%)
ELM	3.73 × 10^−5^	1.37 × 10^−5^	84.64
SVM	1.69 × 10^−4^	5.88 × 10^−5^	80.67
KNN	—	5.00 × 10^−5^	78.66

**Table 6:** Comparison of performance of different classifiers (convolutional networks features).

Names of classifiers	Training time (s)	Time of classification (s)	Recognition rates (%)
ELM	3.82 × 10^−5^	1.24 × 10^−5^	93.04
SVM	1.12 × 10^−4^	4.05 × 10^−5^	82.67
KNN	—	9.73 × 10^−5^	86.67
